# The polypeptide GALNT6 Displays Redundant Functions upon Suppression of its Closest Homolog GALNT3 in Mediating Aberrant O-Glycosylation, Associated with Ovarian Cancer Progression

**DOI:** 10.3390/ijms20092264

**Published:** 2019-05-08

**Authors:** Razan Sheta, Magdalena Bachvarova, Elizabeth Macdonald, Stephane Gobeil, Barbara Vanderhyden, Dimcho Bachvarov

**Affiliations:** 1 Department of Molecular Medicine, Université Laval, Québec, QC G1V 0A6, Canada; razan.sheta.1@ulaval.ca (R.S.); stephane.gobeil@crchul.ulaval.ca (S.G.); 2 CHU de Québec Research Center, Oncology axis Québec, Québec, QC G1V 4G2, Canada; magdalenab3@hotmail.com; 3 Department of Cellular and Molecular Medicine, University of Ottawa, Ottawa, ON K1N 6N5, Canada; ElMacdonald@ohri.ca (E.M.); bvanderhyden@ohri.ca (B.V.); 4 CHU de Québec Research Center, Endocrinology and Nephrology axis Québec, Québec, QC G1V 4G2, Canada

**Keywords:** epithelial ovarian cancer, O-glycosylation, N-acetylgalactosaminyltransferases, VVA lectin, microarrays, intraperitoneal tumor formation

## Abstract

Epithelial ovarian cancer (EOC) represents the most lethal gynecologic malignancy; a better understanding of the molecular mechanisms associated with EOC etiology could substantially improve EOC management. Aberrant O-glycosylation in cancer is attributed to alteration of N-acetylgalactosaminyltransferases (GalNAc-Ts). Reports suggest a genetic and functional redundancy between GalNAc-Ts, and our previous data are indicative of an induction of GALNT6 expression upon GALNT3 suppression in EOC cells. We performed single GALNT3 and double GALNT3/T6 suppression in EOC cells, using a combination of the CRISPR-Cas9 system and shRNA-mediated gene silencing. The effect of single GALNT3 and double GALNT3/T6 inhibition was monitored both *in vitro* (on EOC cells roliferation, migration, and invasion) and *in vivo* (on tumor formation and survival of experimental animals). We confirmed that GALNT3 gene ablation leads to strong and rather compensatory GALNT6 upregulation in EOC cells. Moreover, double GALNT3/T6 suppression was significantly associated with stronger inhibitory effects on EOC cell proliferation, migration, and invasion, and accordingly displayed a significant increase in animal survival rates compared with GALNT3-ablated and control (Ctrl) EOC cells. Our data suggest a possible functional redundancy of GalNAc-Ts (GALNT3 and T6) in EOC, with the perspective of using both these enzymes as novel EOC biomarkers and/or therapeutic targets.

## 1. Introduction

Epithelial ovarian cancer (EOC) is an aggressive disease that is responsible for more cancer deaths among women in the Western world than all other gynecologic malignancies [1,2]. EOC lethality stems from the difficulty in detecting the disease at an early, organ-confined stage, and the lack of effective therapies for the advanced-stages of the disease, which represent the abrogating challenges associated with EOC dissemination [1]. Thus, there is urgent need for new therapeutic targets and a better understanding of the molecular mechanisms involved in EOC etiology.

Glycosylation is the most complex form of all post-translational modifications (PTMs) and is defined by the regulated process of adding lipids and carbohydrates to proteins, as this regulation depends on enzymes and proteases that allow for the diversification of glycoprotein structure and function [3]. Several protein glycosylation types are currently known, the major ones representing the N-linked and the O-linked (mucin) glycosylation [4]. Glycosylation perturbations, including aberrantly glycosylated sugars, affect cellular proliferation, differentiation, migration, invasion, and cell cycle control [5]. The most examined type of O-glycosylation is the mucin type, which is mediated by 20 N-acetylgalactosaminyltransferases (GalNAc-Ts) [6]. Altered expression of O-glycans in cancer is frequently attributed to under- or overexpression of members of the GalNAc-T family [7]; however, few studies have examined the role of different GalNAc-Ts in EOC. We previously identified the GalNAc-T3 (GALNT3) gene as a potential EOC oncogene, highly expressed in advanced disease, as GALNT3 expression was significantly associated with poor outcome [8]. We also demonstrated that GALNT3 could contribute to EOC dissemination through the aberrant glycosylation of different O-glycoproteins, including the MUC1 oncogene [8]. Consecutively, we analyzed alterations in glycoproteins’ expression following GALNT3 gene knockdown (KD) in EOC cells using a metabolic labeling strategy for enrichment and glycoprotein analysis [9]. This glycoproteomics approach led to the identification of numerous O-glycoproteins that were differentially expressed upon GALNT3 KD [9]. Studies on other GalNAc-Ts in EOC were indicative of a role of GALNT14 in promoting EOC cell migration by modulating MUC13 glycosylation [10], and of a role of GALN6 in modifying EGFR O-glycosylation [11].

Although different GalNAc-Ts are differentially expressed within tissues, between cells, and in different patterns at different stages in development and differentiation, it is now clear that a subset of GalNAc-Ts display both distinct and overlapping substrate specificities [12]. Some mammalian GalNAc-T isoforms have been grouped into subfamilies based on their high homology [12]. Evolutionary studies cannot predict why the GalNac-T family is so diverse, and if this diversity can be linked to some degree of redundancy between the genes. This could possibly be related to the seemingly broad and partly overlapping roles of the different GalNAc-Ts in protein O-glycosylation. Data from our previous study showed that there is simultaneous overexpression of four GalNAc-Ts (GALNT3, T6, T9, and T14) in advanced EOC [13]. We have also observed an induction of GALNT6 gene expression following GALNT3 KD in EOC cells, which suggests a possible functional overlap shared by these two enzymes in EOC [13]. Moreover, GALNT6 exhibits analogous oncogenic functions in breast cancer (modulating aberrant O-glycosylation and MUC1 stabilization) [14], similar to observations found by us for GALNT3 in EOC dissemination [8]. 

In this study, we investigated a possible functional redundancy between GALNT3 and GALNT6 in EOC by using both in vitro and in vivo EOC experimental models, as well as the eventual effect of this redundancy on EOC progression.

## 2. Results

### 2.1. GALNT3 Gene Knockout (KO) Triggers GALNT6 Overexpression in EOC Cells

**Figure 1:**
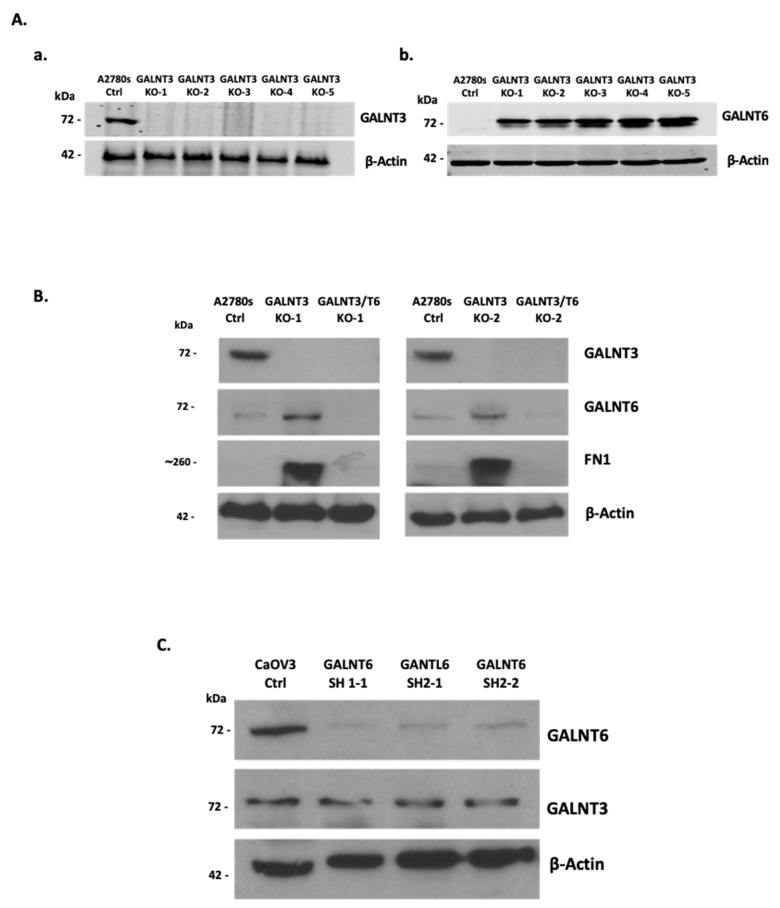
Western blot protein expression analysis of GALNT3 KO and GALNT3/6 KO clones. (**A**) The CRISPR/Cas9 system was used to generate GALNT3 KO (GALNT3 KO) clones in the A2780s cell line. Five clones show complete protein ablation upon GALNT3 KO (**a**). Similarly, Western blot confirms the compensation by GALNT6 in the GALNT3 KO clones (**b**). (**B**) Western blot analysis of the double KO clones (GALNT3/T6 KO-1 and GALNT3/T6 KO-2) generated using the CRISPR/Cas9 system followed by the shRNA system; mock transfected A2780s cells were used as the control clone (A2780s Ctrl). FN1 was also evaluated in these clones, confirming the activity and expression of GALNT6 in the single GALNT3 KO clones. (**C**) Western blot confirmation of shRNA-mediated GALNT6 KD in the CaOV3 cell line, in addition to GALNT3 protein expression analysis in the CaOV3 Ctrl and GALNT6 KD clones. β-actin was used as the loading control.

Initially, we examined for a possible redundant role of GALNT3 and GALNT6 in EOC dissemination using in vitro EOC cellular models. As shown previously [8,15], the A2780s EOC cell line displayed a strong GALNT3 expression and a rather weak GALNT6 expression, and was consequently chosen for our future experiments. GALNT3 KO clones were generated in A2780s cells using clustered regularly interspaced short palindromic repeats associated protein 9 (CRISPR/Cas9) technology (Figure 1(Aa)). Interestingly, all of the GALNT3 KO A2780s clones showed an increase in GALNT6 protein expression (Figure 1(Ab)). Moreover, fibronectin (FN1), previously identified as a specific GALNT6 substrate [15], displayed a strong increase in expression in the GALNT3 KO clones compared with the corresponding control cells (Ctrls) (Figure 1B), which is further indicative of a possible compensatory function of the GALNT6 gene upon GALNT3 KO in EOC cells. 

We also verified that other GalNAc-T members could show altered expression upon GALNT3 KO in the A2780s cells, since GALNT9 and GALNT14 were highly expressed in several EOC cell lines including A2780s, while GALNT2 did not show any expression in all EOC lines tested, as shown previously [13]. However, none of these GalNAc-Ts displayed alterations in protein expression upon GALNT3 KO in A2780s cells (Figure S1A). In order to validate the observed compensatory effect induced by GALNT6 in the GALNT3 clones in the A2780s cell line (Figure 1A), we also used a short hairpin (shRNA)-mediated KD approach to ablate GALNT3 expression in another EOC cell line (SKOV3). We successfully generated two GALNT3 KD clones that showed a strong decrease in GALNT3 protein expression with a corresponding increase in GALNT6 protein expression (Figure S1B). Due to technical difficulties establishing clones using the CRISPR/Cas9 KO approach on the SKOV3 cell line, we decided to focus our work on using the A2780s cell line CRISPR KO clones. 

### 2.2. Effect of Double GALNT3/T6 KO on Protein Glycosylation in EOC Cells

We further generated double GALNT3/T6 gene KOs in the A2780s cell line, as we applied the shRNA approach to target GALNT6 in the GALNT3 KO clones. We initially tested the efficiency of our shRNAs in the CaOV3 EOC cell line, due to the high endogenous GALNT6 expression previously observed in this cell line [13]. Our shRNA-mediated KD approach led to a strong suppression of GALNT6 expression in CaOV3 cells (Figure 1C). However, no differences in GALNT3 protein expression levels were observed upon GALNT6 KD in CaOV3 cells (Figure 1C), possibly due to the relatively strong endogenous expression of GALNT3 in this cell line. 

We next created double GALNT3/T6 KO clones in the A2780s cell line by using the above tested shRNAs to abolish GALNT6 expression in the GALNT3 KO A2780s clones. Consecutive Western blot analyses were indicative of a complete ablation of GALNT6 expression in the selected A2780s clones, which are hereafter referred to as GALNT3/T6 double KO clones (Figure 1B). 

**Figure 2:**
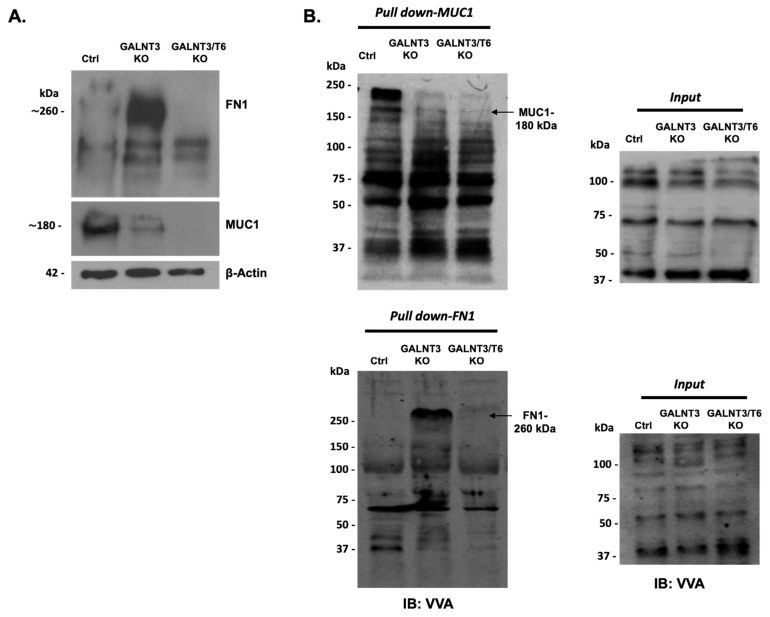
Glycoprotein analysis in GALNT3 KO and GALNT3/T6 KO clones. (**A**) Western blot analysis of FN1 and MUC1 expression in the GALNT3 KO and GALNT3/T6 KO clones compared with Ctrls in the A2780s cell line. β-actin was used as the loading control. (**B**) VVA-lectin-mediated immunoblot analysis of GalNAc-conjugated proteins in protein lysates of the Ctrl, GALNT3 KO, and GALNT3/T6 KO A2780s clones following VVA lectin pull-down assay (pull-down). Upper panel: pull-down MUC1; lower panel: pull-down FN1. Analyses of the crude protein extracts (input) demonstrate comparable total proteins expression levels in the different samples. Arrows indicate the glycosylated protein band.

The effects of single (GALNT3) or double (GALNT3/T6) KO in A2780s cells were further evaluated by analyzing the protein expression and the glycosylation levels of MUC1 and FN1, representing O-glycosylation substrates for these two enzymes. As shown previously [8], a significant reduction in MUC1 protein expression was observed following GALNT3 suppression; however, MUC1 expression was now completely abolished in the GALNT3/T6 double KO clone (Figure 2A). Moreover, FN1 was highly upregulated in the GALNT3 KO clone, while it was absent in both the Ctrl and GALNT3/T6 KO clones (Figure 1B and Figure 2A).

These data were further complemented by *Vicia villosa* (VVA) lectin pull-down assays, as the glycosylated band of MUC1 protein detected by VVA lectin Western blots in the Ctrl clone was slightly reduced in the GALNT3 KO clone, but completely absent in the double GALNT3/T6 clone (Figure 2B). Moreover, the VVA lectin blots examined for FN1 protein expression also showed a strong increase in the FN1 glycosylated band in the GALNT3 KO clone, which was completely absent in both the Ctrl and the GALNT3/T6 double KO clones (Figure 2B). Importantly, no significant differences were observed at the mRNA levels between the Ctrl, GALNT3 KO, and GALNT3/T6 KO clones (Figure S2A,B), indicating that alterations of MUC1 and FN1 protein expression levels are due to glycosylation modifications.

### 2.3. Double GALNT3/T6 KO Results in Stronger Suppression of A2780s Cellular Proliferation, Migration, and Invasion, when Compared with the Single GALNT3 KO

**Figure 3:**
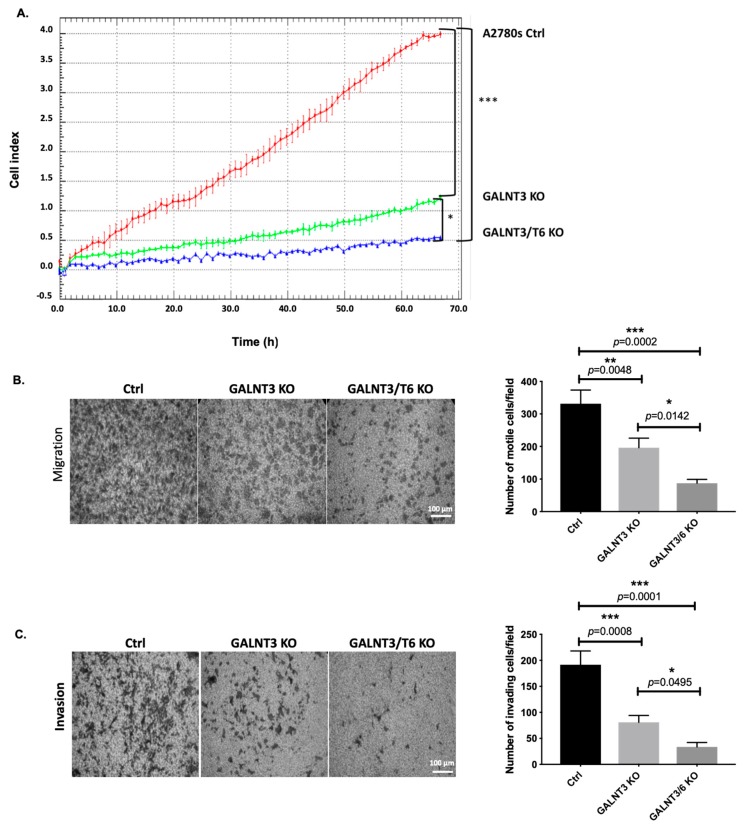
Effect of the GALNT3 KO and the double GALNT3/T6 KO on A2780s cell proliferation, migration and invasion. (**A**) Cell proliferation of GALNT3 KO and GALNT3/T6 KO clones compared with the control clone (Ctrl). Cell index refers to a relative change in electrical impedance representing the number of cells detected on the microelectrodes on the bottom of the xCELLigence wells. (**B**) Cell migration of GALNT3 KO and GALNT3/T6 KO clones compared with the control clone (Ctrl) (scale bar 100 μm). (**C**) Cell invasion of GALNT3 KO and GALNT3/T6 KO clones, as compared with the control clone (Ctrl) (scale bar 100 μm). The corresponding histograms represent quantitative determinations of migration and invasion data obtained by selecting 10 random fields per filter under phase contrast microscopy; results are expressed as number of cell changes (migration and invasion) of GALNT3 KO and GALNT3/T6 KO clones compared with the control clone (Ctrl) (*n* = 3). Data are presented as mean ± SEM and were analyzed by one-way ANOVA followed by a Dunnett’s test for multiple comparisons. A significant association was considered when *p*-values were <0.05. GraphPad style was used for *p*-value presentation; *p*-value symbols refer as follows: ns *p* > 0.05, * *p* ≤ 0.05, ** *p* ≤ 0.01, *** *p* ≤ 0.001, **** *p* ≤ 0.0001.

Multiple functional assays were employed to compare cancer-related phenotypic changes acquired in EOC cells upon knocking out one (GALNT3) or two members (GALNT3/T6) of the GalNAc-T family. One GALNT3 KO A2780s clone (GALNT3 KO) and one GALNT3/T6 double KO (GALNT3/T6 KO) A2780s clone were selected for further functional analyses. The impact of the GALNT3 KO and the double GALNT3/T6 depletion was investigated on A2780s cellular proliferation, migration, invasion and cell cycle control. As expected, the GALNT3 gene ablation led to a sharp significant decrease in A2780s cellular proliferation (represented by the cell index), compared with the Ctrl cells (Figure 3A), and a stronger significant decrease was observed in the double GALNT3/T6 KO clone (Figure 3A). These observations were supported by reduced colony formation upon double GALNT3/T6 suppression compared with the single GALNT3 KO and Ctrls (Figure S3). Additionally, GALNT3 depletion induced G1 cell cycle arrest, and this observation was more prominent when examining the impact of the double GALNT3/T6 KO clone on cell cycle control (Figure S4). Cell cycle analysis could additionally explain the drastic reduction in the proliferation rates of GALNT3 KO and GALNT3/T6 KO cells observed in Figure 3A. Further, knocking out the GALNT3 gene inhibited both the migration and invasion of A2780s cells (Figure 3B,C), but interestingly the number of A2780s cells that passed through the filter in the double GALNT3/T6 KO A2780s clones was significantly lower when compared with both the single GALNT3 KO clone and the Ctrl clone.

### 2.4. Molecular Mechanisms of GALNT3 and GALNT6 Action in EOC Cells

To better understand the molecular mechanisms of GALNT3 and GALNT6 in EOC cells, we employed the Agilent whole human genome 4 × 44K microarrays (containing 44,000 genes) to identify gene expression changes upon GALNT3 KO and GALNT3/T6 KO in A2780s cells. The gene expression patterns of the selected clones were compared against the corresponding Ctrl clone, as all microarray experiments were performed in duplicates, using biological replicas. For all comparisons, a subset of differentially expressed genes were selected by an initial filtering on confidence at *p*-value ≤ 0.05, followed by filtering of expression level (≥1.5 fold). Using these selection criteria, we found 417 upregulated genes and 868 downregulated genes in A2780s cells following GALNT3 KO, while a bigger number of differentially expressed genes were detected in the GALNT3/T6 double KO clone, where 2503 genes were found upregulated and 4553 genes were found downregulated (see GEO submission, GSE104861). 

**Figure 4:**
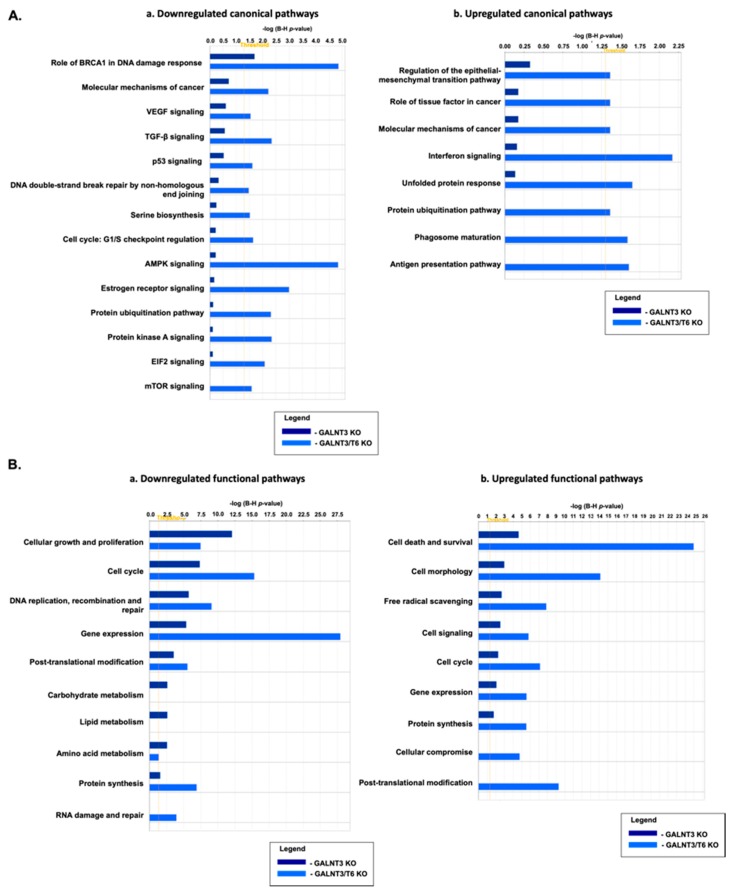
Comparative canonical and functional pathway analysis for a dataset of differentially expressed genes (≥1.5-fold; *p* < 0.05) following GALNT3 KO and GALNT3/T6 KO in A2780s cells. (**A**) (**a**) Comparison analysis of downregulated canonical pathways; (**b**) comparison analysis of upregulated canonical pathways. Top functions that meet a Holm–Bonferroni multiple testing correction *p*-value of 0.05 are displayed. (**B**) (**a**) Comparison analysis of downregulated functional pathways; (**b**) comparison analysis of upregulated functional pathways. Top functions that meet a Holm–Bonferroni multiple testing correction *p*-value of 0.05 are displayed. The bar-charts represent significance of gene enrichment for any given pathway with scores of 2 < z-score < −2.

Consecutive canonical and functional pathway and network analyses generated through Ingenuity Pathway Analysis (IPA) software sustained the cancer-related phenotypic changes observed in A2780s cells following single (GALNT3) gene KO and double (GALNT3/T6) gene KO. Thus, the most significantly downregulated canonical pathways observed in the double gene KO A2780s cell clones, when compared with the single gene KO cell clones, were related to the BRCA1 role in DNA damage response, the molecular mechanisms of cancer, p-53 signaling, and cell cycle regulation, in addition to major cancer-related signaling pathways such as AMPK signaling, and the mTOR signaling pathway (Figure 4(Aa)). Accordingly, upregulated canonical pathways in the double KO clones were predominantly associated with the regulation of the epithelial-mesenchymal transition (EMT) pathway and several protein regulation pathways (Figure 4(Ab)). Successive functional pathway analyses confirmed the above comparisons, and were also supportive of our in vitro functional assays (Figure 4B). Indeed, the most significantly downregulated functional pathways observed when comparing the double GALNT3/T6 gene KO to the single GALNT3 gene KO were related to cell cycle, DNA replication, recombination and repair, and protein synthesis (Figure 4(Ba)), while major upregulated pathways following the double gene KO were related to cell death and survival, cell morphology, and cell signaling (Figure 4(Bb)). Interestingly, the single (GALNT3) KO clones displayed downregulation predominantly in metabolic pathways related to carbohydrate and lipid metabolism, when compared with the double GALNT3/T6 gene KO clones (Figure 4(Ba)). 

**Figure 5:**
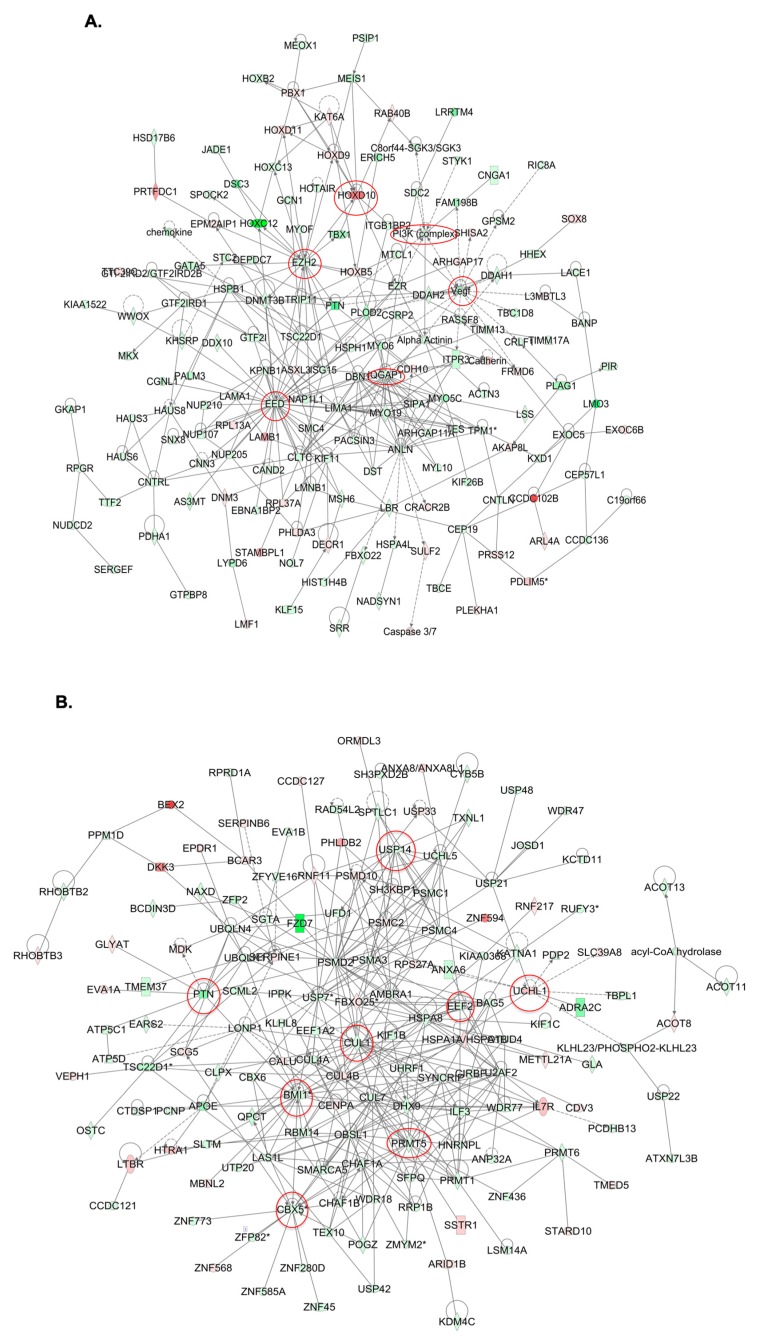
Dynamic gene expression analysis in A2780s cells following both GALNT3 KO and GALNT3/T6 double KO. (**A**) Network analysis of dynamic gene expression in A2780s cells based on the 1.5-fold gene expression list obtained following GALNT3 KO. The five top-scoring networks of up- and downregulated genes were merged and are displayed graphically as nodes (genes/gene products) and edges (the biological relationships between the nodes). (**B**) Network analysis of dynamic gene expression in A2780s cells based on the 1.5-fold gene expression list obtained following GALNT3/T6 double KO. The five top-scoring networks of up- and downregulated genes were merged and are displayed graphically as nodes (genes/gene products) and edges (the biological relationships between the nodes). Intensity of node color indicates the degree of upregulation (red) or downregulation (green). Nodes are displayed using various shapes that represent the functional class of the gene product (square, cytokine, vertical oval, transmembrane receptor, rectangle, nuclear receptor, diamond, enzyme, rhomboid, transporter, hexagon, translation factor, horizontal oval, transcription factor, circle, etc.). Edges are displayed with various labels that describe the nature of the relationship between the nodes: __ binding only, → acts on. Dotted edges represent indirect interaction. Circled nodes in red represent the major gene nodes examined in this study.

Common IPA networks obtained upon merging the five top-scoring networks following both GALNT3 KO and GALNT3/T6 KO were indicative of gene nodes with altered expression succeeding the suppression of these GalNAc-T enzymes in EOC cells. As shown in Figure 5A, GALNT3 KO led to a strong downregulation in gene nodes known to be implicated in EOC tumorigenesis, including VEGF, PI3K complex, and members of the PRC2 complex. Likewise, the top five common IPA networks following GALNT3/T6 suppression were indicative for the downregulation of major gene nodes reported to have important implications in EOC progression, including BMI-1 and members of the PRMT family, in addition to gene nodes showing great promise as EOC therapeutic targets, such as CUL1, PTN, CBX5, and USP14 (Figure 5B). 

To validate microarray results, a panel of differentially expressed genes from both GALNT3 KO and GALNT3/T6 KO microarray data were arbitrarily selected, and their expression was quantified by quantitative PCR (qPCR) upon comparison with the corresponding Ctrls (Figure S5). 

### 2.5. Double GALNT3/T6 Gene KO Reduces EOC Metastasis in vivo

We further investigated if the demonstrated in vitro oncogenic potential of the GALNT3 and GALNT6 genes could be confirmed in vivo, and thus help emphasize their strong implication in EOC tumorigenesis. EOC spreads by intraperitoneal (IP) sloughing, lymphatic invasion, and hematogenous dissemination [16]. Several reports have demonstrated that inoculation of tumor cells through IP injection can best mimic EOC metastasis [17]. We used a similar in vivo approach; thus, Ctrl, GALNT3 KO and GALNT3/T6 KO A2780s clones were IP injected into severe combined immunodeficient (SCID) mice (*n* = 8 Ctrl group, *n* = 8 GALNT3 KO group, and *n* = 8 GALNT3/T6 KO group). Survival and tumor burden analysis was carried out on seven of eight control mice (one mouse was removed due to bowel obstruction, with no sign of disease), eight of eight GALNT3 KO mice and seven of eight GALNT3/T6 KO mice (one mouse was removed due to infection prior to tumor establishment).

Mice injected with Ctrl cells presented with abdominal and liver tumors and tumors forming in the diaphraghm, in addition to several animals presenting with tumors forming in the ovaries. Mice injected with GALNT3 KO cells presented with tumors mostly forming in the lower abdomen, at the point of injection, in addition to tumors arising from the pancreas/omentum, gut mesentery, and the liver. As for mice injected with GALNT3/T6 KO cells, animals presented with tumors arising at the site of injection, as well as tumor formation in the pancreas/omentum and gut mesentary area with normal tissue usually still apparent. 

**Figure 6:**
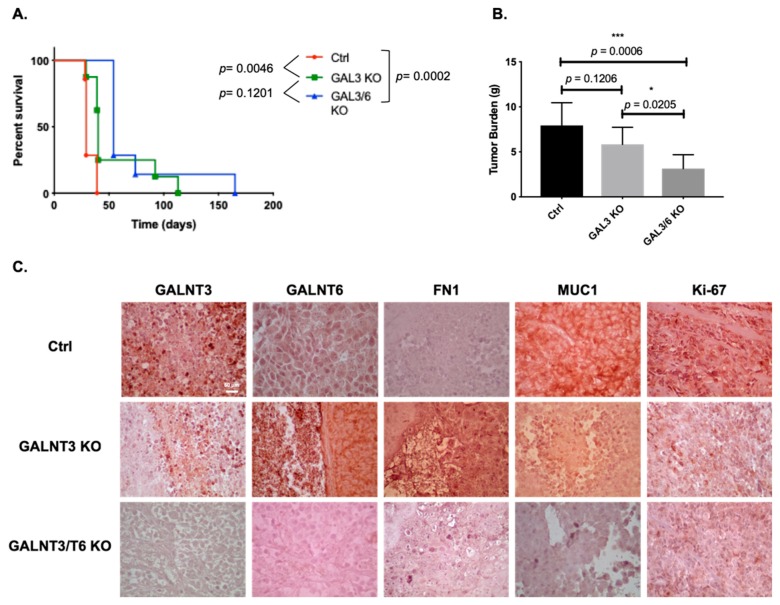
In vivo examination of the effects of GALNT3 and GALNT3/T6 KO in tumor formation and survival in severe combined immunodeficient (SCID) mice. (**A**) Survival curves for mice injected with the Ctrl, GALNT3 KO, and GALNT3/T6 KO EOC cells. Median survival of mice injected with Ctrl cells (32 days, *n* = 7). Survival of mice injected with the GALNT3 KO cells was significantly improved compared with the vector control (*p* = 0.0046, log-rank test), and importantly, mice injected with the GALNT3/T6 KO cells displayed significantly higher survival rates compared with mice injected with Ctrl cells (*p* = 0.0002, log-rank test). (**B**) Tumor weights in SCID mice were measured. Data shown represent the tumor weight averages from seven mice injected with Ctrl cells, eight animals injected with GALNT3 KO cells and seven animals injected with GALNT3/T6 KO cells. Data are presented as mean ± SEM and were analyzed by one-way ANOVA followed by a Dunnett’s test for multiple comparisons. A significant association was considered when *p*-values were <0.05. GraphPad style was used for *p*-value presentation; *p*-value symbols refer as follows: ns *p* > 0.05, * *p* ≤ 0.05, ** *p* ≤ 0.01, *** *p* ≤ 0.001, **** *p* ≤ 0.0001. (**C**) Representative immunohistochemistry images of GALNT3, GALNT6, FN1, MUC1, and Ki-67 expression in tumor tissues extracted from mice injected with the Ctrl, GALNT3 KO, and GALNT3/T6 KO cells at 40× (scale bar 60 μm).

Survival analysis showed that mice injected with Ctrl cells displayed a significantly shorter survival (*p* = 0.0046) than those injected with GALNT3 KO cells, reaching the endpoint on average 32 (+/−1.63 SEM) compared with 54 (+/−9.57 SEM) days post-injection, respectively (Figure 6A). Moreover, the double GALNT3/T6 KO cell line resulted in a significant improvement of survival rate when compared with mice injected with the Ctrl cells (*p* = 0.0002), reaching the endpoint on average 72 (+/−13.54) days post-injection (Figure 6A). No signifincat differences were observed between mice injected with the double GALNT3/T6 KO cell and the GALNT3 KO cells (*p* = 0.1201) (Figure 6A). 

Moreover, there were no significant differences in the tumor burden between mice injected with Ctrl cells and GALNT3 KO cells (*p* = 0.1206), reaching an average tumor mass of 7.9 g (+/−0.83 SEM), compared with 5.8 g (+/−0.59 SEM), respectively (Figure 6B). In comparison, mice injected with the GALNT3/T6 KO cells displayed significantly smaller tumor masses when compared with both the Ctrl and GALNT3 KO groups (*p* = 0.0006 and *p* = 0.0205 respectively), with a mean residual tumor mass of 3.1 g (+/−0.5 SEM) at the endpoint (Figure 6B). These mice were euthanized due to general loss of wellness associated with disseminated disease (particularly in the liver), rather than overall tumor burden. 

Consistent with our previous in vitro data, tumor specimens derived from GALNT3 KO and GALNT3/T6 KO cells showed low immunohistochemical (IHC) staining intensity for the GALNT3 protein (Figure 6C). Moreover, tumors derived from GALNT3 KO cells displayed strong staining for GALNT6 and FN1, and low staining for MUC1 when compared with Ctrl A2780-derived tumors (Figure 6C). Accordingly, GALNT3/T6 KO-derived tumor tissues showed reduced staining intensity for all of GALNT6, FN1, and MUC1 when compared with the Ctrl and GALNT3 KO tissues (Figure 6C). Finally, tumors from both GALNT3 KO and GALNT3/T6 KO-injected mice showed a decrease in staining intensity for the proliferation marker Ki-67 (Figure 6C), which was consistant with our reported in vitro data (Figure 3A). The IHC results were further confirmed by Western blot analysis (Figure S6). These data support our in vitro findings for the role of GALNT3 and GALNT6 in aberrant O-glycosylation in EOC cells.

## 3. Discussion

It is well established that aberrant O-glycosylation plays a very important role in regulating cellular migration/invasiveness during tumorigenesis [5]. These functional characteristics have been reported to depend on the altered expression of different members of the GalNAc-Ts family, as inhibition of one or multiple GalNAc-Ts was shown to either induce or reduce tumor cell invasions and metastasis formation in different cancer types [10,18]. Multiple in vitro studies on GalNAc-Ts suggest that these enzymes display a wide variety of roles in the process of protein O-glycosylation, but their regulation and specified role in normal development and cancer biology have not been profoundly investigated. A number of GalNac-T synthetic deficiencies have been generated in animal model studies, but only a few displayed some effects on animal development or survival. Indeed, in vivo examination of deficiencies in the GalNAc-T genes was reported to cause subtle phenotypic alterations in animal models when monitoring cancer development or metastasis [12]. This has been related to the seemingly broad and partly overlapping roles of the different GalNAc-Ts in protein O-glycosylation. The lack of presentable phenotypic differences is most likely not a result of very subtle changes in the genome, but rather the outcome of effective compensatory mechanisms that have allowed for a decrease in major perturbations acquired from a single gene deletion. It seems that the suppression of one GalNAc-T gene may not be enough to produce detectable phenotypic changes, or to affect the survival of experimental animals [6].

In this study, we closely examined the possible functional overlap of two GalNAc-Ts (GALNT3 and its closest homolog GALNT6), implicated in mediating aberrant O-glycosylation in EOC cells. Both GALNT3 and GALNT6 displayed oncogenic functions in different cancer types; GALNT3 has often been suggested as a possible therapeutic target in pancreatic [19], gastric [20], and colorectal cancers [21]. Similarly, GALNT6 has been extensively studied for its oncogenic role in malignant diseases, such as breast [14], pancreatic [22], and gastric cancer [23]. GALNT6 was also shown to be overexpressed in different EOC histotypes, as it was suggested that in low-grade serous carcinoma, GalNAc-T6 expression may contribute to improved long-term survival [24]. Our recent data were similarly indicative of strong GALNT6 overexpression in EOC samples and, importantly, GALNT6 expression correlated with poor prognosis of high-grade serous EOC patients [13]. Our observations were further confirmed in silico based on the analysis of the GALNT6 expression profiles from publicly available data [13], and upon using the Kaplan–Meier plotter human ovarian cancer data sets [25] for high-grade serous EOC patients, with a follow-up threshold of 60 months (accessible via http://www.kmplot.com/ovar web portal; data not shown). Moreover, increased GALNT3 and GALNTT6 co-expression has been detected in pancreatic [26] and prostate carcinomas [27], and a strong correlation between both GALNT3 and GALNT6 expression has been reported during the different stages of endometriosis [28]. Our previously published data [13] were also indicative of a possible functional overlap of the GALNT3 and GALNT6 enzymes in cancer. Overall, these reports suggest that alterations in GALNT3 expression during malignant transformations could depend on GALNT6 expression levels (and possibly vice versa) in both synergistic and compensatory ways.

Our data strongly indicate an induction of GALNT6 expression in A2780s EOC cells following GALNT3 KO, which is supported by the major increase in FN1 expression in the GALNT3 KO clones. Moreover, cell migration, invasion, proliferation, and cell cycle control were significantly reduced/altered in the double GALNT3/T6 gene KO cells compared with the single GALNT3 gene KO, suggesting that the induction of the GALNT6 expression provided strong backup functional activity in the GALNT3 KO clones. Furthermore, MUC1 protein expression was relatively downregulated in the GALNT3 KO, but displayed much stronger suppression in the GALNT3/T6 KO cells, when both were examined by protein expression analysis and VVA lectin pull-down assays. MUC1 has been previously suggested as a direct substrate of GALNT6 [14], and it appears that the compensatory GALNT6 expression upon GALNT3 KO in EOC cells was sufficient to contribute to the O-glycosylation and stabilization of the MUC1 protein. MUC1 is a glycoprotein that has been reported to play a key role as an oncogene inducing the tumorigenicity of many human cancers [29]. The downregulation of the MUC1 protein, observed in our study upon knocking down both the GALNT3 and GALNT6 genes, emphasizes its possible role in enhancing the malignant phenotype of ovarian cancer. The exact pathway of how MUC1 contributes to many cancer malignant phenotypes has not been clearly identified; however, there are several studies that link MUC1 in inducing major antiapoptotic pathways [30,31], in addition to other studies that confirm MUC1 interaction with canonical pathways associated with cellular transformation and cell growth [32,33]. Thus, our study emphasizes GALNT3 and GALNT6 as druggable EOC targets, since they show potential importance in targeting MUC1 protein expression. 

Global gene expression experiments and consecutive pathway and network analyses confirmed the major effects of GALNT3 KO and GALNT3/T6 double KO on modulating key canonical and functional EOC pathways. Similar to our previously published work [8], GALNT3 KO in A2780s cells resulted in the downregulation of major functional pathways such as cellular growth, cell proliferation and cell cycle. However, our data display compelling evidence to the significance of knocking out both genes (GALNT3 and T6), as our functional and canonical pathway analysis shows more prominent effects on EOC with a higher fold change in major pathways such as cell cycle, DNA replication, and PTM, in addition to reductions in the expression of major genes involved in EOC tumorigenesis. Our results are also in accordance with work recently published by Lin et al. examining the role of GALNT6 in ES-2 ovarian cancer cells [11], as their results confirm the effect of GALNT6 inhibition on the downregulation of various pathways, including cell cycle, cell migration, and major PTMs such as protein phosphorylation [11]. Likewise, our network analyses further support the role of these two GalNAc-Ts in modulating EOC tumorigenesis. Indeed major gene nodes with proven implications in EOC tumorigenesis, including VEGF, PI3K, EZH2, IQGAP1, and HOXD10 [34,35,36,37,38], were downregulated upon GALNT3 ablation. Moreover, multiple gene nodes reported to be involved in EOC progression (such as CUL1 EEF2, PTN, UCHL1, CBX5, USP14, and BMI-1 [39,40,41,42,43,44]) were strongly affected by the GALNT3/T6 double KO. Our gene expression profiling data thus specify many of the putative mechanisms the two genes may play in ovarian carcinogenesis, supporting the functional implications of GALNT3 and GALNT6 in EOC dissemination.

Finally, our in vivo studies undoubtedly support the observed functional redundancy imposed by GALNT6 in EOC cells, as experimental animals injected with double GALNT3/T6 KO EOC clones showed a significant increase in survival compared with those injected with a single GALNT3 gene KO or Ctrl clones, respectively. Our study is the first to report a positive effect on mice survival upon knocking out two GalNAc-Ts, which is suggestive for the potential use of both these genes as EOC therapeutic targets. 

Further studies are necessary to verify possible GALNT3/T6 redundancies in other cancer types, and especially in breast cancer, where GALNT6 has displayed quite similar functions in mediating aberrant O-glycosylation [14], as found by us for GALNT3 in EOC [8]. We also intended to examine if ablation of the GALNT6 gene could similarly invoke GALNT3 overexpression and associated redundant functions in EOC cells; however almost all tested EOC cell lines displayed a strong endogenous GALNT3 expression (with the exception of the OV2008 cell line lacking both GALNT3 and GALNT6 endogenous expression) [8,13]. Thus, the lack of an appropriate cellular model hampered further analyses of possible compensation effects imposed by GALNT3 upon GALNT6 suppression in EOC cells.

In conclusion, our data confirm our and others previous data concerning the oncogenic potentials of GALNT3 and GALNT6 in EOC etiology. Moreover, we provide compelling evidence for the existence of possible functional overlap of these two GalNAc-Ts in EOC, thus highlighting the importance of examining multiple members of GalNAc-Ts for prognostic information for EOC patients. Accordingly, screening for combined GALNT3 and T6 inhibitors could be valuable for the development of novel therapeutic modalities against EOC.

## 4. Materials and Methods

### 4.1. Cell Culture

The EOC cell lines CaOV3 and SKOV3 were purchased from the American Tissue Type Collection (Manassas, VA, USA); the A2780s cell line was a gift from Dr. Benjamin Tsang (Ottawa University, Ottawa, ON Canada) [45]. All cells were maintained at 37 °C and 5% CO_2_ in Dulbecco’s Modified Eagle’s Medium (DMEM; Sigma Aldrich, Oakville, ON Canada) and supplemented with 10% (v/v) fetal bovine serum (FBS; Wisent, Saint-Jean-Baptiste, PQ, Canada) and 1% penicillin/streptomycin (Sigma Aldrich, Oakville, ON Canada). 

### 4.2. CRISPR/Cas9-Mediated GALNT3 Knockout in EOC Cells

CRISPR/Cas9-mediated GALNT3 KO in A2780s cells was performed according to the manufacturer’s guidelines (Santa Cruz Biotechnology, Dallas, TX, USA) as previously described [46]. Twenty-four hours prior to transfection, A2780s cells were plated in a 6-well plate (1.5 × 10^5^ cells per well in 3 mL of antibiotic-free DMEM) and grown to ~70% confluency. We first prepared solution A, which consisted of a plasmid mixture containing: 2 μg of GALNT3 CRISPR/Cas9 KO (sc-407682) (sense: CTCACGTATCCAGATATAAC) and 2 μg GALNT3 homology-directed repair (HDR) plasmids (sc-407682-HDR). Similarly, 2 μg of the Ctrl CRISPR/Cas9 plasmid (sc-418922) was used as a negative control. The plasmid DNA mixture was then added to the Plasmid Transfection Medium (sc-108062) to a final volume of 150 µl. We next prepared solution B, containing 5 μL of UltraCruz Transfection Reagent (sc-395739) diluted in Plasmid Transfection Medium (sc-108062) to a final volume of 150 µl. The plasmid DNA solution (solution A) was then directly added to the UltraCruz Transfection Reagent (solution B) and, prior to transfection, the media in the 6-well plate was replaced with fresh antibiotic-free DMEM. Cells were incubated with the Transfection Reagent complex (solution A + solution B) for 48 h under normal conditions, and media was replaced 48 h post-transfection with fresh medium containing 2 µg/mL puromycin. Media was consecutively replaced every 2–3 days and cell colonies were selected after 10 days incubation. The co-transfection of the CRISPR/Cas9 plasmid with the HDR plasmid led to gene disruption through homologous-directed repair. Stably transfected clones were selected by adding puromycin (0,5 mg/mL). A Western blot was performed to further select colonies with a biallelic knockout of the gene of interest. Monoallelic KOs in our cell population were selected by isolating single cell colonies from cell populations. Clones were verified by Western blot analysis (Figure 1(Aa)). For excision of the puromycin gene, flanked by the LoxP sites, the Cre vector transfection was performed on the positive selected clones co-transfected with CRISPR/Cas9 KO plasmid and HDR plasmid. Selected GALNT3 KO A2780s and Ctrl A2780s clones were plated in a 6-well plate (1.5 × 10^5^ cells per well in 3 mL of antibiotic-free DMEM). Five µl of UltraCruz Transfection Reagent (sc-395739) was added to 3 μg of Cre vector (sc-418923), and the same transfection protocol was performed as described above. Finally, a number of cell clones were tested using Western blot analysis to confirm complete allelic knockouts (Figure 1(Aa)). Selected clones were used for further experimentation after three passages post-puromycin selection. 

### 4.3. shRNA-Mediated GALNT6 KO in EOC Cells

The shRNA-mediated GALNT6 and GALNT3 KD in EOC cells was done as previously described [8]. Two GALNT6 shRNAs cloned into pLKO.1-puro vector (targeting GALNT6 mRNA sequences 5′-GCCATGAACAACCTTAGAGAT-3′ and 5′-GCACAATGTCTACCCAGAGAT-3′) (clone number TRCN0000035489 and TRCN0000035490), one GALNT3 shRNA cloned into pLKO.1-puro vector (targeting GALNT3 mRNA sequences 5′-GCTCTATTCTTCACCTGCAAT-3′) (clone number TRCN0000035454), were retrieved from the Sigma Mission TRC human 1.5 shRNA library. The pLKO.1-puro vector encoding a scramble sequence not matching any mammalian sequence was used for the generation of mock-transduced (control) clones. Cell lines to be infected were plated at a proper density in a 6-well tissue culture plate in 3 mL of rich DMEM containing (a) A2780s cells, seeded at 1 × 10^5^ cells per well, (b) CaOV3 cells, seeded at 1 × 10^5^ cells per well, and (c) SKOV3 cells, seeded at 0.5 × 10^5^ cells per well. Then, 800 μL of viral shRNAs were mixed with fresh DMEM to a final volume of 2 mL, and polybrene (Sigma Aldrich, Oakville, ON Canada) was added to the 2 mL of virus/media for a final concentration of 8 μg/mL. Twenty-four hours post-infection, the virus media was replaced with DMEM. The next day, the media were replaced with 2 mL of DMEM supplemented with the proper concentration of puromycin (2 μg/mL for A2780s cells, 1.5 μg/mL for CaOV3 cells, and 1.5 μg/mL for SKOV3 cells). Cells were transferred to a 10-cm dish for further propagation, and clone selection started when single cells formed sufficiently sized colonies. Colonies were picked and propagated for several weeks and passaged at least twice before using the cells for functional assays. Stable clones with suppressed GALNT6 and GALNT3 expression were validated by Western blot (Figure 1B,C and Figure S1B). 

### 4.4. Western Blotting

Western blot analyses were performed as previously described [13]. Prior to protein extraction, cells were washed with 1× PBS and total protein fraction was extracted by adding 2× Laemmli buffer (Sigma Aldrich, Oakville, ON Canada) and incubated at 95 °C for 20 min for DNA denaturation. Tumor and control tissues from the in vivo experiments were homogenized and sonicated in RIPA buffer [50 mM Tris (pH 7.4), 150 mM NaCl, 0.5% sodium deoxycholate, 0.1% SDS, 1% Triton x- 100] containing protease and phosphatase inhibitors, then samples were incubated on ice for 15 min. After centrifugation at 13,300 rpm for 15 min at 4 °C, the supernatant was taken and 20–30 μg of the protein was used for sample preparation. Protein measurement was performed using BCA Protein Assay (Thermo Scientific Pierce, Waltham, MA USA), following the manufacturer’s guidelines. Consecutively, 15–25 μg of protein per well was loaded in 10% or 12% SDS–PAGE gels. Gels were run at 130 V for 75 min. Membranes were blocked in 4% milk in TBST for 1 h at room temperature (RT), the membranes were then incubated with the appropriate primary antibody at 4 °C overnight. Membranes were incubated with horseradish peroxidase-conjugated secondary antibody for 1 h at RT and detected with an ECL Western Blotting Substrate (Thermo Fisher Scientific, PQ, Canada). Western blot protein bands were analyzed for statistical analysis using ImageJ software (ImageJ 1.51s (100)) (National Institutes of Health, Bethesda, MD, USA). List of antibodies used: anti-GALNT3 (AP9208C, 1:1000, Abgent, San Diego, CA, USA), anti-MUC1 (sc-7313, 1:1000, Santa Cruz Biotechnology, Dallas, TX, USA), anti-FN1 (sc-69681, 1:1000, Santa Cruz Biotechnology), anti-GALNT6 (ab151329, 1:500, Abcam, Toronto, ON, Canada), anti-β-Actin (sc-517582, 1:3000, Santa Cruz BiotechnologyDallas, TX, USA), goat-anti-rabbit HRP conjugated (ab6721, 1:1000, Abcam Toronto, ON, Canada), and goat-anti-mouse HRP conjugated (ab6789, 1:1000, Abcam Toronto, ON, Canada). 

### 4.5. Functional Assays

#### 4.5.1. Cell Proliferation Assay

Cellular proliferation of GALNT3 CRISPR/Cas9 KO clones, GALNT3-T6 (shRNA/CRISPR/Cas9 KO clones), and Ctrl cells was monitored with the xCELLigence Real-Time Cell Analyzer (RTCA) (Roche Applied Science and ACEA Biosciences, Mississauga, ON, Canada), as previously described [8]. Cells in complete DMEM (100 μL) were seeded in triplicates in the xCELLigence plates (1.5 × 10^3^ cells per well) and cellular proliferation was monitored for 70 h. The output electrical inputs were converted to a cell index every 1 h. The cell index refers to a relative change in electrical impedance representing the number of cells detected on the microelectrodes on the bottom of the xCELLigence wells. Cell index profiles were normalized at 2 h and the slope of growth curves was determined and quantified. 

#### 4.5.2. Colony Formation Assay

GALNT3 CRISPR/Cas9 KO clones, GALNT3-T6 (shRNA/CRISPR/Cas9 KO clones), and Ctrl cells were seeded at 500 cells per 60-mm culture dish. After 14 days, plates were washed twice in PBS, fixed with cold methanol, stained with Coomassie Brilliant Blue (Sigma Aldrich, Oakville, ON Canada) for 5 min, washed with water, and air dried. The number of colonies was determined by imaging with ImageJ. The experiments were performed in triplicates. 

#### 4.5.3. Migration Assay

Cell migration assays were performed as previously described [8]. Briefly, GALNT3 CRISPR/Cas9 KO clones, GALNT3-T6 (shRNA/CRISPR/Cas9 KO clones), and Ctrl cells were seeded into the upper inserts of Boyden chambers (Corning; Sigma Aldrich, Oakville, ON Canada) in 0.1% FBS-containing medium at a density of 2.5 × 10^4^ per well; 600 μL of 1% FBS-containing medium was added to the lower chamber as a chemoattractant. After 24 h incubation at 37 °C in 5% CO_2_, the cells were fixed with cold methanol and stained with trypan blue solution. Cells on the upper surface of the filter were removed with cotton buds. Migrated cells on the underside of the filter were photographed and counted by phase contrast microscopy, by selecting 10 random fields per filter (at 40× magnification). The experiments were performed in triplicates. 

#### 4.5.4. Invasion Assay

Cell invasion was assayed in a similar way [8], as the 5-mm pore polycarbonate filters were coated with 40 μL of Matrigel at a concentration of 0.5 mg/mL (BD Biosciences, San Jose, CA, USA). Here, 600 μL of NIH3T3-conditioned medium was added in the lower chamber as a chemoattractant. Invading cells on the underside of the filter were photographed and counted by phase contrast microscopy by selecting 10 random fields per filter (at 40× magnification). The experiments were performed in triplicates. Differences between GALNT3, GALNT3/T6 KO, and the corresponding Ctrl clones were determined by a Student’s *t*-test, where *p* < 0.05 was considered significant; the same statistical test was performed for all functional assays.

#### 4.5.5. Flow Cytometry-Cell Cycle Analysis

Flow cytometer analysis was performed as previously described [45]. Briefly, 7.5 × 10^4^ A2780s Ctrl, GALNT3 KO, and GALNT3/T6 KO cells were treated with 20 mM hydroxyurea (Sigma Aldrich, Oakville, ON Canada) for synchronization at the G_1_/S boundary. After 16 h of incubation, cells were washed once with PBS and resuspended in 1 mL of complete media (time 0). Then, cells were harvested by trypsinization at 0, 6, 12, and 24 h, washed three times with ice-cold PBS and fixed with ice-cold 95% ethanol overnight. Cells were washed with PBS (3×) and incubated with propidium iodide (PI) solution, containing 50 μg/mL of PI (Sigma Aldrich, Oakville, ON Canada) dissolved in 0.1% sodium citrate (Sigma), 1 mg/mL RNAse (Sigma Aldrich, Oakville, ON Canada), and 0.1% Nonidet P40 (Sigma Aldrich, Oakville, ON Canada); samples were left in the dark at room temperature for 60 min. Flow cytometric analysis was performed on a Beckman Coulter EPICS XL-MCL analyzer. The cell-cycle phase distribution was calculated from the resultant DNA using cell QuesPro software.

### 4.6. Semi-Quantitative RT-PCR (sqRT-PCR) and Quantitaive PCR (qPCR)

Analysis of gene expression in transfected GALNT3 and GALNT3/T6 KO clones and corresponding mock-transfected clones (Ctrl) was performed by RT-PCR and RT-qPCR as previously described [8]. Total RNA was extracted from cells using an RNeasy Mini Kit (Qiagen, Germantown, MD, USA), according to the manufacturer’s protocol. For RT-PCR, 2 μg of RNA was used for reverse transcription. The cDNA was then subjected to PCR amplification using the primers listed in Table S1. mRNA data was detected on a 1% agarose gel with Safe-Green (Abm). Relative mRNA levels of target genes were normalized to GAPDH, which was used as a loading control. For RT-qPCR, RNA was reverse-transcribed into cDNA using Superscript III transcriptase, according to the manufacturer’s protocol (Invitrogen; Thermo Fisher Scientific, Waltham, MA, USA). RT-qPCR was performed using the SYBR Green PCR Master Mix (Applied Biosystems; Thermo Fisher Scientific Waltham, MA, USA) on a ROTOR GENE real-time PCR machine (Corbett Robotics, Qiagen, Germantown, MD, USA). Primers were designed as previously shown [8]; all primers for qPCR are listed in Table A1. Relative quantification of RNA expression was calculated using the 2^−ΔΔCq^ method [47]. The GUSB gene was used as an internal standard. Each sample was tested in triplicate. 

### 4.7. VVA Lectin Pull-Down Assay for O-Glycosylated (GalNAc-conjugated) Proteins

Protein lysates from cells were extracted and measured as described in the Western blot section, and 600 µg of cell lysate protein was incubated for 3 h at 4 °C with 4 µg of biotinylated lectin VVA (EY Laboratories). Upon adding of 20 µl of streptavidin-agarose (Sigma Aldrich, Oakville, ON Canada), samples were incubated for an additional 2 h at 4 °C with rotation. Lectin/glycoprotein complexes were collected by brief centrifugation (1400 rpm, 5 min), and washed 3× with lysis buffer, followed by one wash with PBS. Glycoproteins were released from the complexes by boiling in 30–50 µl SDS–PAGE sample buffers (5 min). The glycoproteins were resolved by SDS–PAGE as described in the Western blot section, then immunoblotted to detect MUC1 (sc-7313, 1:1000, Santa Cruz Biotechnology, Dallas, TX, USA) and FN1 (sc-69681, 1:1000, Santa Cruz Biotechnology, Dallas, TX, USA) protein expression.

### 4.8. Gene Expression Profiling and Data Analysis

Gene expression analysis was carried out as previously described [46]. Total RNA was extracted from two GALNT3 KO clones, two GALNT3/T6 KO clones, and their corresponding Ctrls (Ctrl). Cyanine-labeled cRNA from the clones suppressing GALNT3 and GALNT3/T6 in the A2780s cells were mixed with the same amount of reverse-color cyanine-labeled cRNA from their corresponding Ctrl clones and hybridized on the Agilent Whole Human Genome microarrays. Pathway and network analyses were completed using IPA software (see http://www.Ingenuity.com). The microarray data have been deposited to the GEO database (http://www.ncbi.nlm.nih.gov/geo/; accession No GSE104861; access on May 07, 2019).

### 4.9. IP Tumor Formation in Mice, and IHC Analysis

Animal experiments were carried out at the University of Ottawa and conform with or exceed standards outlined in the Canadian Council on Animal Care (CCAC) and the Animals for Research Act, RSO 1990, Chapter c. A.22. Six-week-old female mice (*n* = 24; 20 g +/−2; CB17SCID; CB17/Icr-Prkdcscid/IcrIcoCrl strain code 236, Charles River) were randomly assigned to conventional microisolator cages with plastic huts and corn cob bedding in groups of three upon arrival. Mice were maintained in a dedicated room for immunity-compromised mice (21 °C, 40%–60% humidity, 12/12 h light/dark cycle). A commercial rodent diet (2018 Teklad Global 18% Protein Rodent Diet, Harlan Laboratories, Indianapolis, ID, USA) along with acidified water was available ad libitum. Housing, food, and water were autoclaved, and all animal manipulations were carried out in a certified ESCO-type A2 BSC hood, following a two-person dirty/clean protocol. Following a 1-week acclimation period, mice were randomly allocated to three treatment groups (*n* = 8 per group; Ctrl A2780s cells, GALNT3 KO, or GALNT3/T6 KO A2780s cells). IP injections (1 × 10^7^ cells in 250 µl of PBS) were carried out cage by cage, with each cage housing three mice, one mouse per treatment group. Wellness assessments, carried out by the animal care staff on a daily basis, and necropsies of endpointed mice were blinded. End of wellness criteria included: body condition score (BCS), signs of respiratory distress, signs of pain, and presence of palpable tumors or abdominal distension impeding mobility. Daily assessments were made by trained staff, and mouse endpoints were called based on a combination of the above clinical signs. Mice in this study were ended primarily due to abdominal distension, which was a direct result of tumor burden, leading to respiratory distress. Euthanization was carried out by inhalation of carbon dioxide using ancare static microisolators (regulator pressure of 12 PSI and flow of 1.5 LPM), followed by cervical dislocation and immediate tissue harvesting and processing. 

IHC analyses of the expression of different markers (GALNT3, GALNT6, MUC1, FN1, Ki-67) in tumor tissues derived from the experimental animals were performed as previously described [46], and all retrieval methods were performed using Tris-EDTA buffer (10 mM Tris base, 1 mM EDTA solution, pH 6.0) using a pressure cooker. Briefly, tissue sections were deparaffinized and rehydrated in graded alcohols, then incubated with blocking serum for 20 min. Following treatment with 3% H_2_O_2_ for 10 min to quench the endogenous peroxidase activity, sections were incubated with the primary antibody overnight at 4 °C. List of antibodies used: anti-GALN T3 (AP9208C, 1:100, Abgent), anti-MUC1 (sc-7313, 1:50, Santa Cruz Biotechnology Dallas, TX, USA), anti-FN1 (sc-69681, 1:100, Santa Cruz Biotechnology Dallas, TX, USA), anti-GALNT6 (ab151329, 1:75, Abcam Toronto, ON, Canada), and anti-Ki-67 (sc-23900, 1:100, Santa Cruz Biotechnology Dallas, TX, USA). Incubation and detection with SignalStain 3,3’-diaminoben-zidine (DAB) Substrate kit (IDetect universal Mouse Kit hrP-DAB; ID Labs; Sigma Aldrich, Oakville, ON Canada) were done according to the manufacturer’s instructions. Sections were then counter-stained with hematoxylin. Images were acquired using a Leica Confocal Scope (TCS SP5 x; Leica Microsystems, Wetzlar, Germany). 

### 4.10. Statistical Analysis

Prism software (Version 7.0d) was used to determine the statistical significance of differences in the means of experimental groups from Western blots and functional assays used in the study. All data are presented as mean ± SEM and were analyzed by Student’s unpaired two-tailed *t*-tests or by one-way ANOVA followed by a Dunnett’s test for multiple comparisons. All experiments were performed in triplicates. A Kaplan–Meier plotter was used to analyze survival of animal groups used in the study, with a log-rank test to represent survival significance. A significant association was considered when *p*-values were <0.05. GraphPad style was used for *p*-value presentation, if not listed on graphs; *p*-value symbols refer to the following: ns *p* > 0.05, * *p* ≤ 0.05, ** *p* ≤ 0.01, *** *p* ≤ 0.001, **** *p* ≤ 0.0001. 

## Supplementary Materials

Supplementary materials can be found at https://www.mdpi.com/1422-0067/20/9/2264/s1.

## Abbreviations

CRISPR	clustered regularly interspaced short palindromic repeats
Cas9	CRISPR associated protein 9
EMT	Epithelial-mesenchymal transition
EOC	Epithelial ovarian cancer
GalNAc-Ts	N-acetylgalactosaminyltransferases
GEO	Gene Expression Omnibus
HDR	Homology directed repair
IHC	Immunohistochemistry
IPA	Ingenuity Pathway Analysis
KD	Knockdown
KO	Knockout
PTM	Post-translational modification
qPCR	Quantitative PCR
RTCA	Real-Time Cell Analyzer
SCID	Severe combined immunodeficient
shRNA	Short hairpin RNA
sqRT-PCR	Semi-quantitative RT-PCR
VVA	*Vicia villosa*

## Appendix A

**Table A1:** Primers used, and quantitative PCR (qPCR) and semi-quantitative PCR (sq-PCR).

		qPCR Primers		
Gene	Primer	Primer Sequence	Product Size (bp)	Genbank Accession #
*GALNT3*	Forward	5′- CAATCAAGGAGGCAAACCAT -3′	266	NM_004482.3
	Reverse	5′- GCTCTCCATTTGCTGAAAGG -3′		
*GALNT6*	Forward	5′- CACCTTGGCGCTTACGAGA -3′	71	NM_007210.3
	Reverse	5′- CCTGGGAATCTGAACAACCCTC -3′		
*MUC1*	Forward	5′- ATCTCATTGCCTTGGCTGTC -3′	216	NM_001018016.2
	Reverse	5′- CCACTGCTGGGTTTGTGTAA -3′		
*FN1*	Forward	5′- GAGAGAAGTGGGACCGTCAG -3′	165	NM_001306129.1
	Reverse	5′- TGGCACCGAGATATTCCTTC -3′		
*BMP2*	Forward	5′- CCTTTTACTGCCACGGAGAA -3′	215	NM_001200.3
	Reverse	5′- ACAACCCTCCACAACCATGT -3′		
*CFH*	Forward	5′- TGGAAGATGGGATCCAGAAG -3′	243	NM_000186.3
	Reverse	5′- TGAGGTGGTTGTGAACATGG -3′		
*CD36*	Forward	5′- TGGCTGTGTTTGGAGGTATTC -3′	349	NM_000072.3
	Reverse	5′- AAGTTGTCAGCCTCTGTTCCA -3′		
*ZFHX4*	Forward	5′- TCAAGTTTGTGCAGCACCTC -3′	207	NM_024721.4
	Reverse	5′- CACGGGATCCTGTCTTCACT -3′		
*THSD7A*	Forward	5′- GCTGGGGCATTTGTGTTACT -3′	209	NM_015204.2
	Reverse	5′- ATTTGTTGTGGCCTCTGGAC -3′		
*CDKN2A*	Forward	5′- ATATGCCTTCCCCCACTACC -3′	231	NM_000077.4
	Reverse	5′- CCCCTGAGCTTCCCTAGTTC -3′		
*TIMP1*	Forward	5′- AATTCCGACCTCGTCATCAG -3′	230	NM_003254.2
	Reverse	5′- TGCAGTTTTCCAGCAATGAG -3′		
*HGF*	Forward	5′- CTGGTTCCCCTTCAATAGCA -3′	168	NM_000601.5
	Reverse	5′- CTCCAGGGCTGACATTTGAT -3′		
*PCDH9*	Forward	5′- ATGGCAACTCTGATCCCAAC -3′	268	NM_001318372.1
	Reverse	5′- CGGTCATTGAACTGGTTCCT -3′		
*ID4*	Forward	5′- TCCGAAGGGAGTGACTAGGA -3′	152	NM_001546.3
	Reverse	5′- CCGAGCCCAACAATTGAC -3′		
*MMP10*	Forward	5′- AATGAGTTCTGGGCCATCAG -3′	151	NM_002425.2
	Reverse	5′- ATTTGTCCGCTGCAAAGAAG -3′		
*MMP3*	Forward	5′- TCATTTTGGCCATCTCTTCC -3′	155	NM_002422.4
	Reverse	5′- GGGAAACCTAGGGTGTGGAT -3′		
*TGFBI*	Forward	5′- CTGGTGCGGCTAAAGTCTCT -3′	222	NM_000358.2
	Reverse	5′- CGCTGATGCTTGTTTGAAGA -3′		
*MAL2*	Forward	5′- ATCCCTGCATGATTTGCATT -3′	164	NM_052886.2
	Reverse	5′- GAGTGTTACGGTCGCCATCT -3′		
*GREM1*	Forward	5′- GGTATTTGGGTTGAAAGAATTT -3′	424	NM_001191322.1
	Reverse	5′- AAAATACACAACAAATCACATTTTC -3′		
*IFI16*	Forward	5′- CCTTTCACACTTGTGGCTGA -3′	143	NM_001206567.1
	Reverse	5′- ACCTCAAACACCCCATTCAC -3′		
*CASP4*	Forward	5′- GCTTTCTGCTCTTCCACACC -3′	160	NM_001225.3
	Reverse	5′- CATCTGGCTGCTCAAATGAA -3′		
*GUSB*	Forward	5′- ATACGTGGTTGGAGAGCTCATT -3′	187	NM_000181.3
	Reverse	5′- CTTGGCTACTGAGTGGGGATAC -3′		
*18S*	Forward	5′-AACCCGTTGAACCCCATT-3′	119	NR_003278
	Reverse	5′-CCATCCAATCGGTAGTAGCG-3′		

Note: Forward and reverse primers were designed to recognize different exons of the target gene. The primer positions are based on the reference sequence of the target genes in the Genbank. Expression of 18S and GUSB were used as control for sample normalization in quantification of the gene of interest expression by real-time RT-PCR analysis.
