# Supplementary material for: The polypeptide GALNT6 Displays Redundant Functions upon Suppression of its Closest Homolog GALNT3 in Mediating Aberrant O-Glycosylation, Associated with Ovarian Cancer Progression
Source: Int J Mol Sci. 2019 May 8;20(9):2264. doi: 10.3390/ijms20092264 (PMC6539655; doi:10.3390/ijms20092264)
Supplement: Supplementary file 1 [file ijms-20-02264-s001.pdf]

International Journal of
Molecular Sciences
MDPI

Supplementary Figures

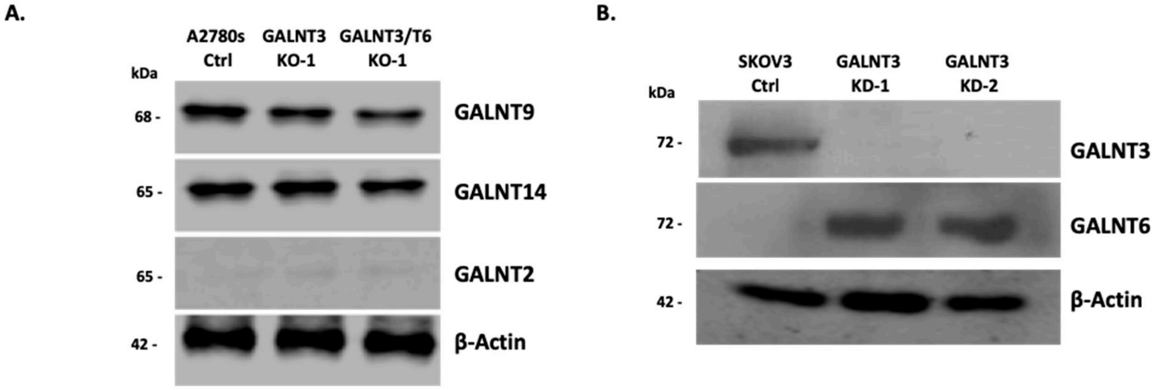

Supplementary Figure S1. Western blot protein expression analysis of GALNT3 KO and GALNT3/6 KO clones. (A) Western blot protein expression analysis of GALNT3 KO and GALNT3/6 KO clones. Western blot examination of GALNT protein expression (GALNT2, GALNT9 and GALNT14) in both the GALNT3 KO and GALNT3/T6 KO clones. (B) Western blot protein expression analysis of GALNT3 KO and GALNT3/6 KO clones. The shRNA gene KD system was used to generate GALNT3 KD (GALNT3 KD) clones in the SKOV3 cell line. Two clones show complete protein ablation upon GALNT3 KD. Similarly, the Western blot confirms the compensation by GALNT6 in the GALNT3 KD clones.

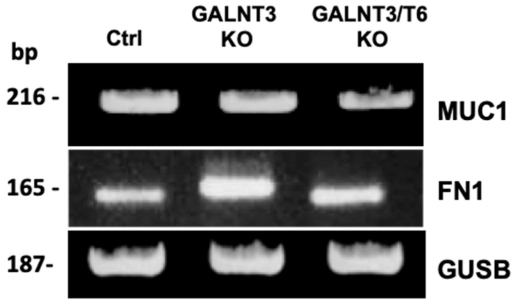

Supplementary Figure S2. Semi-quantitative RT-PCR analysis of GALNT3 KO and GALNT3/6 KO clones. MUC1 and FN1 mRNA levels in the Ctrl clone, GALNT3 KO and GALNT3/T6 KO clones; the GUSB gene was used as an internal control.

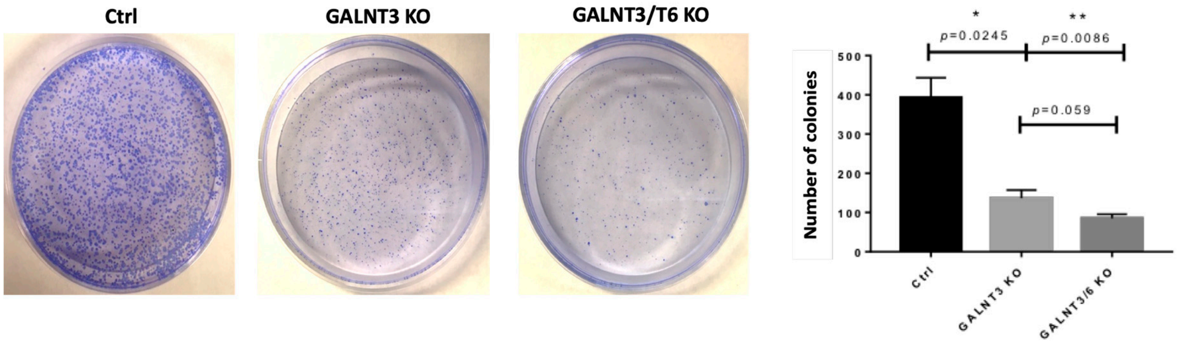

Supplementary Figure S3. Functional analysis of GALNT3 KO and GALNT3/6 KO clones. Representative images of colony forming assays following GALNT3 KO and GALNT3/T6 KO. The corresponding bar graphs represent quantitative determinations of the colony formation assays

obtained by determining the percentage of the area covered by crystal violet stained cell colonies, and the intensity of the staining of the colonies per plate. Results are expressed as number of colony differences between the GALNT3 KO and GALNT3/T6 KO clones compared to the Ctrl clone. Differences in colony numbers were determined by a Student's t-test; error bars denote mean ± SEM (n = 3); *indicates statistical significance ($p < 0.05$).

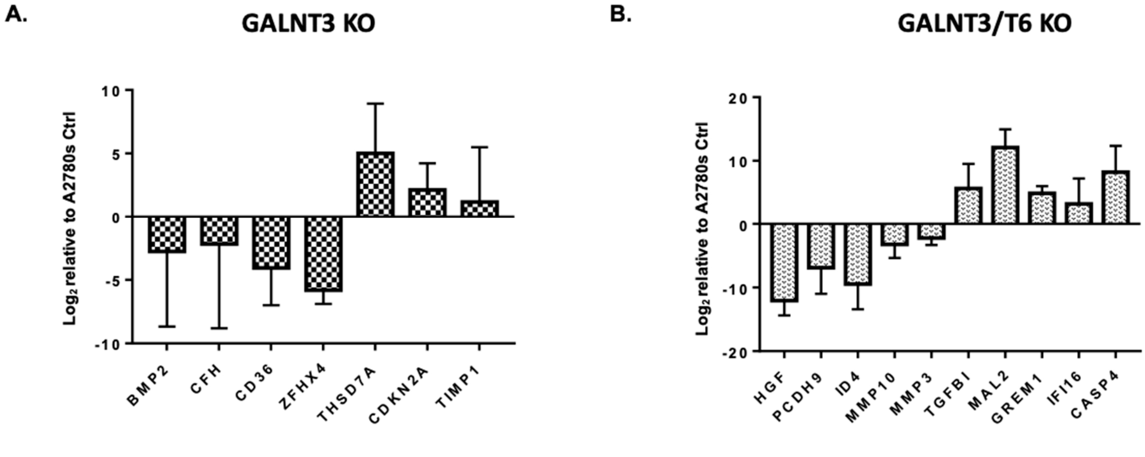

Supplementary Figure S4. Microarray validation of GALNT3 KO and GALNT3/6 KO clones. (A) Bar graphs presentation of the differential expression of selected genes in A2780s cells following GALNT3 KO, compared to Ctrl A2780s cells. (B) Bar graphs presentation of the differential expression of selected genes in A2780s cells following GALNT3/T6 KO, compared to Ctrl A2780s cells. The relative copy number was calculated based on the target gene/18S ribosomal RNA ratio. Values more than or equal to 1 represent gene upregulation and less than 1 display gene downregulation.

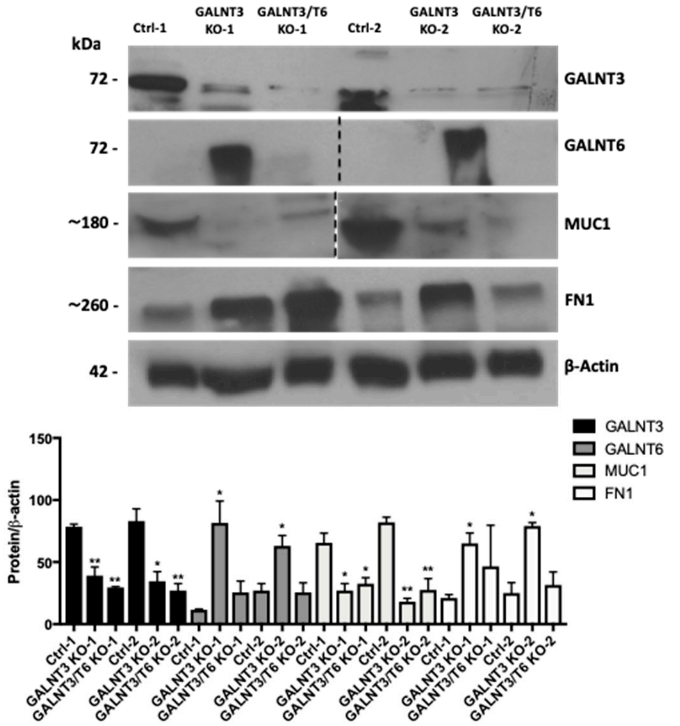

Supplementary Figure S5. Western blot analysis of protein expression from animal model data. Western blot analysis of protein expression (GALNT3, GALNT6, FN1, and MUC1) from tumor tissues of several SCID mice injected with clones used in the study. Histograms representing protein

expression levels compared to the Ctrl sample as normalized against 6-actin (n = 3), data are presented as %. Dashed lines indicate placement of membranes after grouping into the same image.

© 2019 by the authors. Submitted for possible open access publication under the terms and conditions of the Creative Commons Attribution (CC BY) license (http://creativecommons.org/licenses/by/4.0/).
